# Supplementary material for: Differences in stability of seed-associated microbial assemblages in response to invasion by phytopathogenic microorganisms
Source: PeerJ. 2016 Apr 11;4:e1923. doi: 10.7717/peerj.1923 (PMC4830237; doi:10.7717/peerj.1923)
Supplement: Supplemental Information 1 [file peerj-04-1923-s001.docx]

## Supplemental Information

Article title: **Differences in stability of seed-associated microbial assemblages in response to invasion by phytopathogenic microorganisms**

Authors: Samir Rezki^1^, Claire Campion^2^, Beatrice Iacomi-Vasilescu^3^, Anne Préveaux^1^, Youness Toualbia^2^, Sophie Bonneau^1^, Martial Briand^1^, Emmanuelle Laurent^5^, Gilles Hunault^4^, Philippe Simoneau^2^, Marie-Agnès Jacques^1^ and Matthieu Barret^1*^

**Table S1: Primers and probes used in this study**

**Table S2: Summary of the different sequencing runs performed in this study**

This table summarizes information regarding the runs performed with the MiSeq sequencing platform. The number of paired read, quality sequences, operational taxonomic units (OTUs), abundant operational taxonomic units (aOTUs) and sequences belonging to aOTUs are indicated in each column.

**Table S3: Changes in relative abundance of bacterial aOTUs following pathogen transmission**

Columns depicted: affiliation of aOTUs (as assessed with 16S rRNA gene sequences) at the lowest taxonomic level, relative abundance of each aOTU in different samples and the LDA score associated to each treatment.

**Table S4: Changes in relative abundance of bacterial aOTUs following pathogen transmission**

Columns depicted: affiliation of aOTUs (as assessed with *gyrB* sequences) at the lowest taxonomic level, relative abundance of each aOTU in different samples and the LDA score associated to each treatment.

**Table S5: Changes in relative abundance of fungal aOTUs following pathogen transmission**

Columns depicted: affiliation of aOTUs (as assessed with ITS1 sequences) at the lowest taxonomic level, relative abundance of each aOTU in different samples and the LDA score associated to each treatment.

**Table S6: Properties of environment specific networks**

**Figure S1: Estimation of bacterial and fungal abundance on seeds**

Quantification of bacterial (A) and fungal (B) abundance on seeds was performed by qPCR. Black lines represent the copy number of 16S rRNA and ACT genes used to quantify bacterial and fungal abundances in the different seed samples. Black lines represent the median and the grey could represent the density of distribution. Changes between conditions are considered as significant at a *P* ≤ 0.01 (ANOVA with post hoc Tukey’s HSD test).

**Figure S2: Richness and diversity of seed samples observed with abundant and rare OTUs**

Microbial richness (A, B and C) and diversity (D, E and F) were estimated with OTUs obtained with 16S rRNA gene (A and D), *gyrB* (B and E) and ITS1 sequences (C and F). Richness and diversity associated to uncontaminated seeds (C2013 and C2014), seeds contaminated with *Xcc* (X2013 and X2014) and seeds contaminated with *Ab* (A2013 and A2014) were compared. Each sample is represented by a green line, while black line represents the median. The grey area represents the density of distribution. Letters a, b and c denote significant changes between conditions considered at a *P*-value ≤ 0.01 (as assessed by ANOVA with post hoc Tukey’s HSD test).

**Figure S3: Structure of seed-associated bacterial assemblages according to 16S rRNA gene sequences**

Hierarchical clustering of seed samples (*y* axis) is based on Bray-Curtis dissimilarity measure. The type of samples is represented by gradual color changes: light blue for controls, medium blue for seeds contaminated with *Xcc* and dark blue for seeds contaminated with *Ab*. Only abundant OTUs (threshold of 1% in relative abundance) are represented in the heatmap. These aOTUs are clustered by their co-occurrence patterns (*x* axis). According to analysis of similarity, a significant clustering of *Ab* seed samples was observed (*p* < 0.001).

**Figure S4: Structure of seed-associated bacterial assemblages according to *gyrB* sequences**

Hierarchical clustering of seed samples (*y* axis) is based on Bray-Curtis dissimilarity measure. The type of samples is represented by gradual color changes: light blue for controls, medium blue for seeds contaminated with *Xcc* and dark blue for seeds contaminated with *Ab*. Only abundant OTUs (threshold of 1% in relative abundance) are represented in the heatmap. These aOTUs are clustered by their co-occurrence patterns (*x* axis). According to analysis of similarity, a significant clustering of Ab seed samples was observed (*p* < 0.001).

**Figure S5: Changes in relative abundance of microbial taxa**

Relative abundance of bacterial and fungal orders according to 16S rRNA gene (A), *gyrB* (B) and ITS1 (C). Taxonomic affiliation of bacterial aOTUs was performed with the RDP database for 16S rRNA gene, with an in-house *gyrB* database (Barret et al 2015) and with the UNITE database for ITS1.

**Figure S6: Correlations networks between bacterial aOTUs**

Correlation networks between bacterial taxa are based on 16S rRNA gene sequences obtained in uncontaminated seeds (A), seeds contaminated with *Xcc* (B), and contaminated with *Ab* (C).Correlations between aOTUs were calculated with the Sparse Correlations for Compositional data algorithm. Each node represents an aOTUs, which is colored according to its taxonomic affiliation (family-level). Edges represent correlations between the nodes they connect with blue and orange colors indicating negative and positive inferred correlation, respectively. Only correlations with pseudo *p-*value ≤ 0.001 were represented in the network using the R package qgraph.

**Table S1: Primers and probes used in this study**

| **Name** | **Sequence (5' - 3')** | **Reference** |
| --- | --- | --- |
| Zup4F | GCCGGTACGGATGCAGAGCG | This study |
| Zup4R | GCCAGGGTGCATAGGCCACG | This study |
| 926F | AACTCAAAGGAATTGACGG | Lane *et al.* 1991 |
| 1062R | CTCACRRCACGAGCTGAC | Allen *et al.* 2005 |
| AbraDHN1-Tq-F | CACAACCCGCCCTTATCAAA | This study |
| AbraDHN1-Tq-R | CGAGCGGCACGTTCATG | This study |
| TaqMan MGB probe | TGATGCACGTTTGTCTC | This study |
| ACT 512-F | ATGTGCAAGGCCGGTTTCGC | Carbone and Kohn 1999 |
| ACT 783-R | TACGAGTCCTTCTGGCCCAT | Carbone and Kohn 1999 |
| 515F | GTGCCAGCMGCCGCGGTAA | Caporaso *et al.* 2011 |
| 806R | GGACTACVSGGGTATCTAAT | Caporaso *et al.* 2011 |
| gyrB_aF64 | MGNCCNGSNATGTAYATHGG | Barret *et al.* 2015 |
| gyrB_aR353 | ACNCCRTGNARDCCDCCNGA | Barret *et al.* 2015 |
| ITS1F | CTTGGTCATTTAGAGGAAGTAA | Gardes and Bruns 1993 |
| ITS2 | GCTGCGTTCTTCATCGATGC | White *et al.* 1990 |

**Table S2: Summary of the different sequencing runs performed in this study**

| **molecular marker** | **pairs of reads** | **quality sequences** | **OTUs** | **aOTUs (> 0.1% of sequences per sample)** | **Sequences belonging to aOTUs** |
| --- | --- | --- | --- | --- | --- |
| 16S rRNA gene (V4 region) | 7,870,622 | 5,826,763 | 7,550 | 83 | 5,710,255 |
| *gyrB* | 24,355,191 | 20,904,297 | 25,284 | 321 | 20,400,609 |
| ITS1 | 8,799,598 | 7,495,411 | 5,792 | 146 | 7,277,014 |

This table summarizes information regarding the runs performed with the MiSeq sequencing platform. The number of paired read, quality sequences, operational taxonomic units (OTUs), abundant operational taxonomic units (aOTUs) and sequences belonging to aOTUs are indicated in each column.

**Table S3: Changes in relative abundance of bacterial aOTUs following pathogen transmission**

| **OTUNumber** | **Bacterial Taxa** | **%C2013** | **%X2013** | **%A2013** | **%C2014** | **%X2014** | **%A2014** | **LDA score (A vs C)** | **LDA score (X vs C)** |
| --- | --- | --- | --- | --- | --- | --- | --- | --- | --- |
| Otu00004 | Xanthomonas | 1,0 | 2,9 | 1,4 | 2,5 | 12,9 | 4,2 | ns | 4,5 |
| Otu00007 | Chryseobacterium | 0,5 | 0,9 | 0,2 | 5,4 | 4,6 | 2,4 | -4,1 | ns |
| Otu00008 | Enterobacteriaceae | 0,1 | 0,1 | 0,1 | 0,2 | 0,0 | 0,1 | -2,8 | -3,8 |
| Otu00010 | Rhizobium | 1,1 | 1,1 | 0,6 | 1,8 | 1,6 | 1,2 | -3,4 | ns |
| Otu00011 | Pedobacter | 0,1 | 0,2 | 0,2 | 1,3 | 0,8 | 0,4 | -3,4 | ns |
| Otu00020 | Pseudomonas | 0,1 | 0,1 | 0,1 | 0,1 | 0,0 | 0,1 | -2,8 | -3,7 |
| Otu00026 | Enterobacteriales | 0,0 | 0,0 | 0,0 | 0,0 | 0,0 | 0,0 | ns | -3,9 |
| Otu00028 | Gammaproteobacteria | 0,0 | 0,0 | 0,0 | 0,0 | 0,0 | 0,1 | ns | -4,1 |
| Otu00041 | Enterobacteriales | 0,0 | 0,0 | 0,0 | 0,0 | 0,0 | 0,0 | 2,7 | ns |
| Otu00043 | Chryseobacterium | 0,0 | 0,0 | 0,0 | 0,1 | 0,3 | 0,0 | -2,7 | ns |

Columns depicted: affiliation of aOTUs (as assessed with 16S rRNA gene sequences) at the lowest taxonomic level, relative abundance of each aOTU in different samples and the LDA score associated to each treatment.

**Table S4: Changes in relative abundance of bacterial aOTUs following pathogen transmission**

| **OTUNumber** | **Bacterial Taxa** | **%C2013** | **%X2013** | **%A2013** | **%C2014** | **%X2014** | **%A2014** | **LDA score (A vs C)** | **LDA score (X vs C)** |
| --- | --- | --- | --- | --- | --- | --- | --- | --- | --- |
| Otu00003 | Erwinia tasmaniensis | 2,27 | 2,11 | 3,17 | 5,19 | 4,76 | 6,36 | ns | -3,8 |
| Otu00005 | Pseudomonas fluorescens | 3,23 | 0,13 | 1,50 | 8,62 | 0,09 | 0,01 | -4,5 | -4,5 |
| Otu00012 | Pseudomonas fluorescens | 2,69 | 3,52 | 3,28 | 1,00 | 1,12 | 0,41 | ns | 3,7 |
| Otu00013 | Pseudomonas fluorescens | 0,00 | 1,13 | 2,05 | 0,07 | 0,13 | 0,08 | 3,7 | 3,5 |
| Otu00017 | Pseudomonas fluorescens | 0,91 | 0,93 | 1,73 | 0,25 | 0,07 | 1,56 | 3,7 | ns |
| Otu00018 | Rhizobium | 0,11 | 0,13 | 0,05 | 0,09 | 0,20 | 0,05 | -2,8 | ns |
| Otu00024 | Pseudomonas | 0,31 | 0,11 | 0,06 | 0,31 | 0,32 | 0,10 | -3,3 | -3,4 |
| Otu00030 | Pantoea agglomerans | 0,00 | 0,00 | 0,00 | 0,03 | 0,02 | 0,02 | -3,2 | ns |
| Otu00034 | Firmicutes | 0,16 | 0,20 | 0,07 | 0,19 | 0,04 | 0,00 | -2,9 | ns |
| Otu00037 | Pseudomonas poae | 1,01 | 1,10 | 0,10 | 0,00 | 0,01 | 0,08 | -3,0 | 3,4 |
| Otu00038 | Pseudomonas fluorescens | 0,00 | 0,17 | 0,17 | 0,20 | 0,33 | 1,83 | 3,7 | ns |
| Otu00039 | Xanthomonas campestris | 0,06 | 0,31 | 0,02 | 0,07 | 2,07 | 0,13 | ns | 3,8 |
| Otu00040 | Pseudomonas fluorescens | 0,62 | 1,01 | 0,13 | 0,34 | 0,63 | 0,16 | -3,2 | ns |
| Otu00059 | Enterobacter cancerogenus | 0,09 | 0,25 | 0,50 | 0,01 | 0,27 | 0,01 | 3,3 | 3,3 |
| Otu00062 | unclassified | 0,03 | 0,26 | 0,08 | 0,30 | 0,08 | 0,05 | -3,0 | ns |
| Otu00066 | Rhizobiaceae | 0,01 | 0,11 | 0,09 | 0,03 | 0,18 | 0,01 | ns | 3,5 |
| Otu00087 | Rhizobium | 0,14 | 0,04 | 0,01 | 0,06 | 0,11 | 0,00 | -2,8 | ns |
| Otu00150 | Pseudomonas | 0,29 | 0,02 | 0,17 | 0,00 | 0,03 | 0,14 | 2,9 | ns |
| Otu00276 | unclassified | 0,00 | 0,00 | 0,00 | 0,00 | 0,03 | 0,01 | ns | 3,4 |
| Otu00600 | Pseudomonas fluorescens | 0,00 | 0,07 | 0,00 | 0,00 | 0,03 | 0,00 | ns | 3,3 |

Columns depicted: affiliation of aOTUs (as assessed with *gyrB* sequences) at the lowest taxonomic level, relative abundance of each aOTU in different samples and the LDA score associated to each treatment.

**Table S5: Changes in relative abundance of fungal aOTUs following pathogen transmission**

| **OTUNumber** | **Fungal Taxa** | **%C2013** | **%X2013** | **%A2013** | **%C2014** | **%X2014** | **%A2014** | **LDA score (A vs C)** | **LDA score (X vs C)** |
| --- | --- | --- | --- | --- | --- | --- | --- | --- | --- |
| OTU0003 | Alternaria sect. Alternata | 13,2 | 10,8 | 3,4 | 9,1 | 7,9 | 1,0 | -4,6 | ns |
| OTU0013 | Dioszegia | 0,0 | 0,0 | 0,0 | 0,2 | 0,2 | 0,0 | -3,3 | -2,6 |
| OTU0020 | Alternaria | 0,2 | 0,1 | 0,0 | 0,2 | 0,2 | 0,0 | -3,6 | ns |
| OTU0030 | Gibellulopsis | 0,7 | 0,9 | 0,2 | 0,8 | 0,2 | 0,1 | -3,6 | ns |
| OTU0045 | Alternaria | 0,0 | 0,0 | 0,0 | 0,0 | 0,0 | 0,0 | -3,7 | -2,6 |
| OTU0053 | Sordariomycetes | 0,5 | 1,0 | 0,1 | 0,2 | 0,1 | 0,1 | -3,1 | ns |
| OTU0068 | Alternaria | 0,0 | 0,0 | 0,0 | 0,1 | 0,0 | 0,0 | -3,8 | -2,6 |
| OTU0113 | Ramularia coccinea | 0,0 | 0,0 | 0,0 | 0,2 | 0,0 | 0,0 | -3,3 | -2,9 |
| OTU0155 | Cryptococcus | 0,7 | 0,6 | 0,3 | 0,5 | 0,5 | 0,1 | -3,3 | ns |
| OTU0172 | Bulleromyces | 0,1 | 0,1 | 0,1 | 0,2 | 0,4 | 0,0 | -3,1 | ns |
| OTU0241 | Basidiomycota | 1,8 | 3,3 | 2,0 | 3,2 | 2,5 | 1,1 | -3,8 | ns |
| OTU0259 | Alternaria | 2,0 | 1,4 | 0,1 | 1,3 | 1,5 | 0,0 | -3,9 | ns |
| OTU0265 | Alternaria sect. Infectoriae | 0,0 | 0,0 | 0,0 | 0,0 | 0,0 | 0,0 | -3,8 | ns |
| OTU0285 | Gibberella | 1,9 | 1,5 | 0,4 | 0,3 | 1,9 | 0,0 | -3,4 | 3,7 |
| OTU0309 | Sporobolomyces | 0,7 | 1,2 | 0,9 | 0,5 | 0,3 | 0,1 | -3,0 | ns |
| OTU0315 | Basidiomycota | 0,1 | 0,1 | 0,0 | 0,1 | 0,1 | 0,0 | -3,4 | ns |
| OTU0332 | Ascomycota | 0,0 | 0,1 | 0,0 | 0,0 | 0,0 | 0,0 | ns | 2,6 |
| OTU0339 | Basidiomycota | 0,0 | 0,0 | 0,0 | 0,0 | 0,0 | 0,0 | -3,6 | ns |
| OTU0346 | Erysiphe cruciferarum | 0,0 | 0,0 | 0,0 | 0,0 | 0,0 | 0,0 | -3,4 | ns |
| OTU0347 | Alternaria sect. Infectoriae | 0,0 | 0,0 | 0,0 | 0,0 | 0,1 | 0,0 | -3,7 | ns |
| OTU0362 | Filobasidiaceae | 0,3 | 0,7 | 0,7 | 0,1 | 0,2 | 0,0 | ns | 3,1 |
| OTU0389 | Ampelomyces | 0,0 | 0,0 | 0,0 | 0,1 | 0,0 | 0,0 | -3,5 | ns |
| OTU0436 | Alternaria sect. Japonicae | 0,0 | 0,0 | 0,0 | 0,1 | 0,0 | 0,3 | ns | -2,7 |
| OTU0443 | Alternaria sect. Brassicicola | 0,0 | 0,1 | 1,6 | 0,3 | 0,0 | 1,2 | 3,8 | -3,1 |
| OTU0446 | Alternaria sect. Brassicicola | 0,2 | 0,7 | 57,0 | 2,1 | 0,1 | 79,4 | 5,5 | ns |
| OTU0449 | Alternaria brassicae | 1,4 | 2,8 | 0,9 | 7,2 | 2,9 | 1,2 | -4,4 | ns |
| OTU0473 | Filobasidium | 0,0 | 0,0 | 0,0 | 0,1 | 0,1 | 0,0 | -3,5 | ns |
| OTU0515 | Alternaria | 1,9 | 1,4 | 0,6 | 1,8 | 1,9 | 0,3 | -3,9 | ns |
| OTU0536 | Alternaria sect. Infectoriae | 1,5 | 1,4 | 0,7 | 1,6 | 2,0 | 0,4 | -3,8 | ns |
| OTU0567 | Bensingtonia | 0,0 | 0,0 | 0,0 | 0,1 | 0,1 | 0,0 | -3,5 | ns |
| OTU0589 | Cladosporium cucumerinum | 0,0 | 0,0 | 0,0 | 0,0 | 0,0 | 0,0 | -3,9 | ns |
| OTU0622 | Filobasidium | 0,2 | 0,3 | 0,2 | 0,6 | 0,6 | 0,1 | -3,4 | ns |
| OTU0640 | Alternaria | 2,2 | 2,3 | 1,0 | 0,4 | 0,6 | 0,1 | -3,1 | 3,6 |
| OTU0646 | Alternaria sect. Infectoriae | 43,5 | 38,7 | 9,0 | 33,5 | 43,2 | 2,6 | -5,2 | ns |
| OTU0648 | Sporobolomyces roseus | 0,1 | 0,1 | 0,0 | 0,1 | 0,0 | 0,0 | -3,6 | -2,8 |
| OTU0724 | Cladosporium cucumerinum | 14,5 | 16,8 | 8,0 | 19,2 | 17,4 | 3,9 | -4,8 | ns |
| OTU0737 | Cryptococcus | 0,2 | 0,2 | 0,1 | 0,2 | 0,2 | 0,0 | -3,3 | ns |
| OTU0751 | Basidiomycota | 0,0 | 0,0 | 0,0 | 0,0 | 0,0 | 0,0 | -3,8 | ns |
| OTU0757 | Alternaria sect. Alternata | 0,3 | 0,2 | 0,1 | 0,4 | 0,3 | 0,0 | -3,6 | -2,7 |
| **OTUNumber** | **Fungal Taxa** | **%C2013** | **%X2013** | **%A2013** | **%C2014** | **%X2014** | **%A2014** | **LDA score (A vs C)** | **LDA score (X vs C)** |
| OTU0766 | Alternaria sect. Infectoriae | 0,0 | 0,0 | 0,0 | 0,0 | 0,1 | 0,0 | -3,7 | ns |
| OTU0799 | Mycosphaerellaceae | 1,6 | 1,5 | 0,8 | 1,8 | 1,5 | 0,7 | -3,7 | ns |
| OTU0801 | Fusarium oxysporum | 0,2 | 0,0 | 0,0 | 0,1 | 2,1 | 0,0 | -3,1 | ns |
| OTU0819 | Fusarium equiseti | 0,1 | 0,1 | 0,0 | 0,0 | 0,0 | 0,0 | -4,3 | ns |
| OTU0884 | Alternaria sect. Porri | 0,0 | 0,0 | 0,4 | 0,1 | 0,0 | 0,2 | ns | -2,8 |
| OTU0905 | Alternaria | 1,2 | 0,8 | 0,1 | 0,9 | 1,0 | 0,0 | -3,8 | ns |
| OTU0906 | Alternaria | 0,0 | 0,1 | 2,2 | 0,4 | 0,0 | 1,7 | 3,9 | ns |
| OTU0907 | Alternaria brassicae | 0,1 | 0,1 | 0,0 | 0,4 | 0,3 | 0,0 | -3,5 | ns |
| OTU0908 | Alternaria | 0,0 | 0,0 | 0,0 | 0,1 | 0,1 | 0,0 | -3,5 | -2,6 |
| OTU0909 | Alternaria | 0,9 | 1,2 | 0,1 | 2,5 | 2,0 | 0,0 | -4,0 | ns |
| OTU0912 | Alternaria | 0,4 | 0,4 | 0,0 | 0,1 | 0,2 | 0,0 | -3,6 | 2,9 |
| OTU0914 | Alternaria | 0,4 | 0,3 | 0,2 | 0,3 | 0,4 | 0,1 | -3,4 | ns |
| OTU0916 | Alternaria | 1,7 | 1,1 | 0,1 | 0,9 | 1,1 | 0,0 | -3,8 | ns |
| OTU0917 | Alternaria | 0,0 | 0,0 | 0,2 | 0,0 | 0,0 | 0,0 | 3,3 | ns |
| OTU0918 | Alternaria | 0,1 | 0,1 | 0,0 | 0,0 | 0,0 | 0,0 | -3,6 | ns |
| OTU0919 | Alternaria | 0,7 | 0,6 | 0,1 | 0,1 | 0,3 | 0,0 | -3,3 | 3,1 |
| OTU0922 | Alternaria | 0,2 | 0,2 | 0,0 | 0,0 | 0,0 | 0,0 | -3,6 | 2,7 |
| OTU0930 | Alternaria | 0,2 | 0,3 | 0,6 | 0,8 | 0,3 | 0,3 | ns | -3,2 |
| OTU0931 | Alternaria | 0,3 | 0,5 | 0,0 | 1,5 | 1,1 | 0,0 | -3,8 | ns |
| OTU0932 | Alternaria | 0,4 | 0,4 | 0,1 | 0,1 | 0,1 | 0,0 | -3,3 | 2,9 |
| OTU0933 | Alternaria sect. Infectoriae | 0,0 | 0,0 | 0,0 | 0,0 | 0,1 | 0,0 | -3,6 | ns |
| OTU0934 | Alternaria sect. Infectoriae | 0,1 | 0,0 | 0,0 | 0,2 | 0,3 | 0,0 | -3,7 | ns |
| OTU0937 | Alternaria sect. Brassicicola | 0,0 | 0,0 | 1,0 | 0,1 | 0,0 | 0,8 | 3,7 | -2,8 |
| OTU0938 | Alternaria sect. Infectoriae | 0,0 | 0,0 | 0,0 | 0,0 | 0,0 | 0,0 | -3,4 | ns |
| OTU0939 | Alternaria sect. Infectoriae | 0,2 | 0,2 | 0,1 | 0,3 | 0,4 | 0,0 | -3,4 | ns |
| OTU0947 | unclassified | 0,3 | 0,6 | 0,4 | 0,4 | 0,3 | 0,1 | -3,3 | ns |
| OTU0948 | Alternaria | 0,3 | 0,4 | 0,0 | 0,8 | 0,5 | 0,0 | -3,6 | -3,2 |
| OTU0949 | Alternaria | 0,0 | 0,0 | 0,0 | 0,0 | 0,0 | 0,0 | -3,6 | ns |
| OTU0950 | Alternaria | 0,3 | 0,2 | 0,0 | 0,0 | 0,0 | 0,0 | -3,8 | 2,7 |
| OTU4326 | Alternaria | 0,0 | 0,0 | 0,0 | 0,0 | 0,0 | 0,0 | -3,7 | ns |
| OTU4413 | Alternaria brassicae | 0,0 | 0,0 | 0,0 | 0,0 | 0,0 | 0,0 | -3,8 | -2,7 |
| OTU4482 | Alternaria | 0,0 | 0,0 | 0,0 | 0,0 | 0,0 | 0,0 | ns | -2,8 |
| OTU4553 | Alternaria | 0,0 | 0,0 | 0,1 | 0,0 | 0,0 | 0,1 | 3,4 | -2,7 |
| OTU5086 | Alternaria | 0,0 | 0,0 | 0,0 | 0,1 | 0,0 | 0,0 | -3,8 | -2,8 |
| OTU5220 | Alternaria | 0,0 | 0,0 | 0,2 | 0,0 | 0,0 | 0,0 | 3,4 | ns |

Columns depicted: affiliation of aOTUs (as assessed with ITS1 sequences) at the lowest taxonomic level, relative abundance of each aOTU in different samples and the LDA score associated to each treatment.

**Table S6: Properties of environment specific networks**

| **Parameter** | **C 16S network** | **A 16S network** | **X 16S network** | **C gyrB network** | **A gyrB network** | **X gyrB network** | **C ITS1 network** | **A ITS1 network** | **X ITS1 network** |
| --- | --- | --- | --- | --- | --- | --- | --- | --- | --- |
| number of samples | 56 | 46 | 46 | 56 | 46 | 46 | 56 | 46 | 46 |
| number of nodes | 19 | 16 | 15 | 100 | 109 | 89 | 91 | 62 | 82 |
| number of edges | 52 | 32 | 42 | 206 | 182 | 150 | 942 | 398 | 932 |
| number of modules | 3 | 3 | 4 | 28 | 28 | 35 | 1 | 3 | 2 |
| median nb of connectivity | 3 | 2 | 2 | 1 | 1 | 1 | 10 | 5 | 9.5 |
| maximal degree | 6 | 4 | 7 | 6 | 4 | 5 | 26 | 22 | 33 |


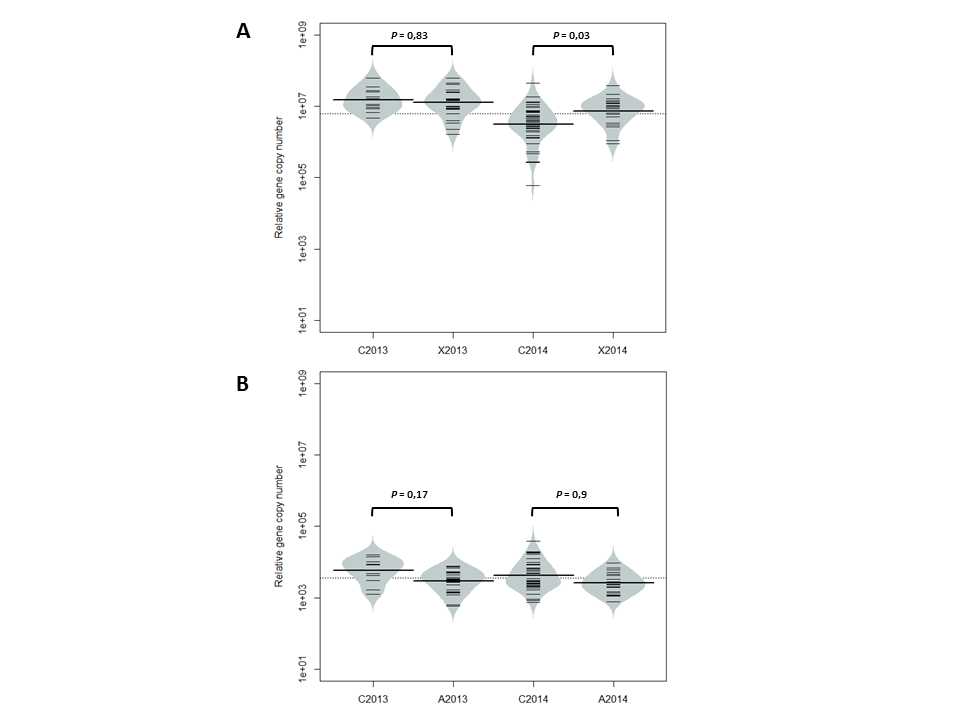


**Figure S1: Estimation of bacterial and fungal abundance on seeds**

Quantification of bacterial (A) and fungal (B) abundance on seeds was performed by qPCR. Black lines represent the copy number of 16S rRNA and ACT genes used to quantify bacterial and fungal abundances in the different seed samples. Black lines represent the median and the grey could represent the density of distribution. Changes between conditions are considered as significant at a *P* ≤ 0.01 (ANOVA with post hoc Tukey’s HSD test).


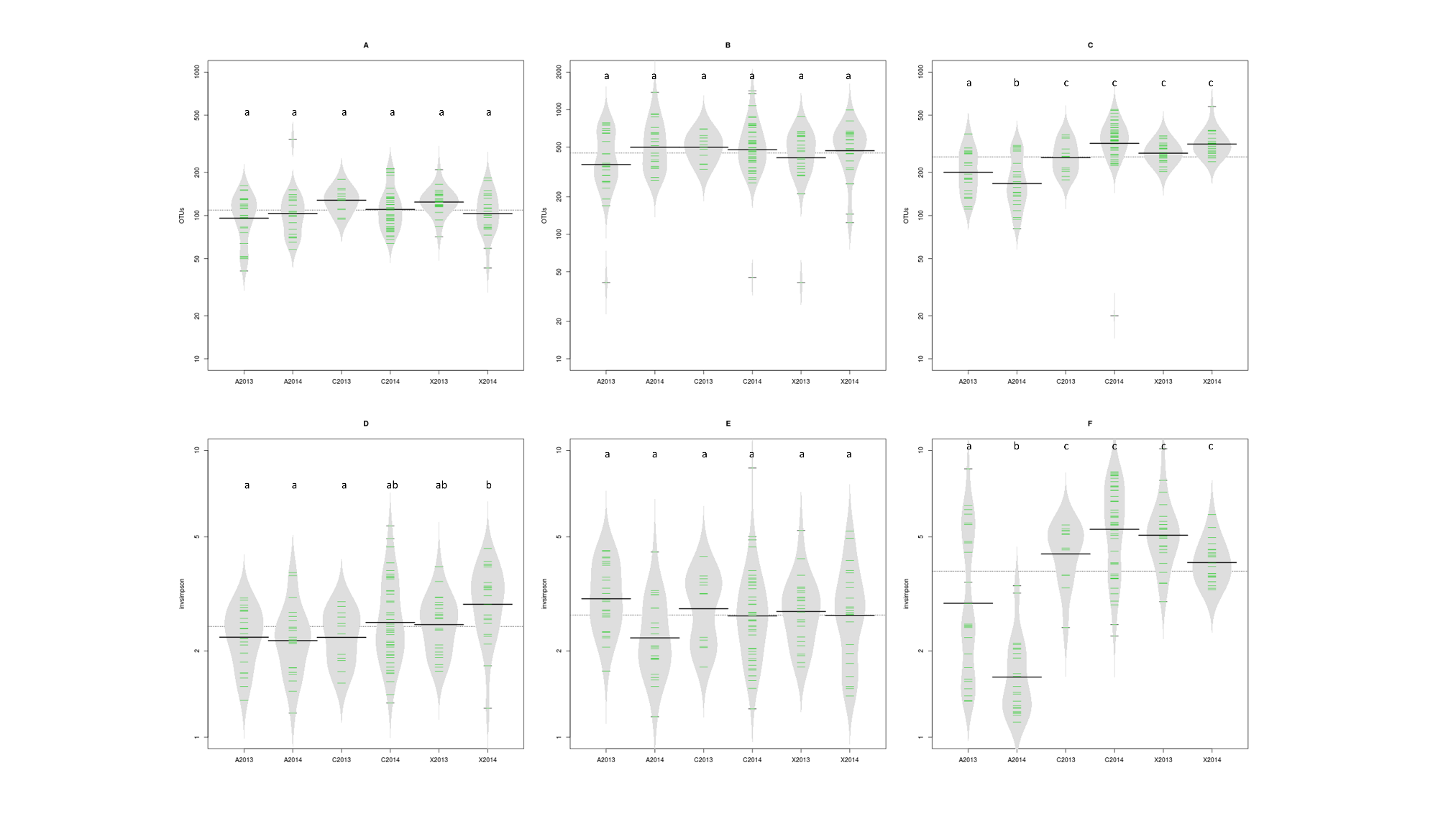


**Figure S2: Richness and diversity of seed samples observed with abundant and rare OTUs**

Microbial richness (A, B and C) and diversity (D, E and F) were estimated with OTUs obtained with 16S rRNA gene (A and D), *gyrB* (B and E) and ITS1 sequences (C and F). Richness and diversity associated to uncontaminated seeds (C2013 and C2014), seeds contaminated with *Xcc* (X2013 and X2014) and seeds contaminated with *Ab* (A2013 and A2014) were compared. Each sample is represented by a green line, while black line represents the median. The grey area represents the density of distribution. Letters a, b and c denote significant changes between conditions considered at a *P*-value ≤ 0.01 (as assessed by ANOVA with post hoc Tukey’s HSD test).

**
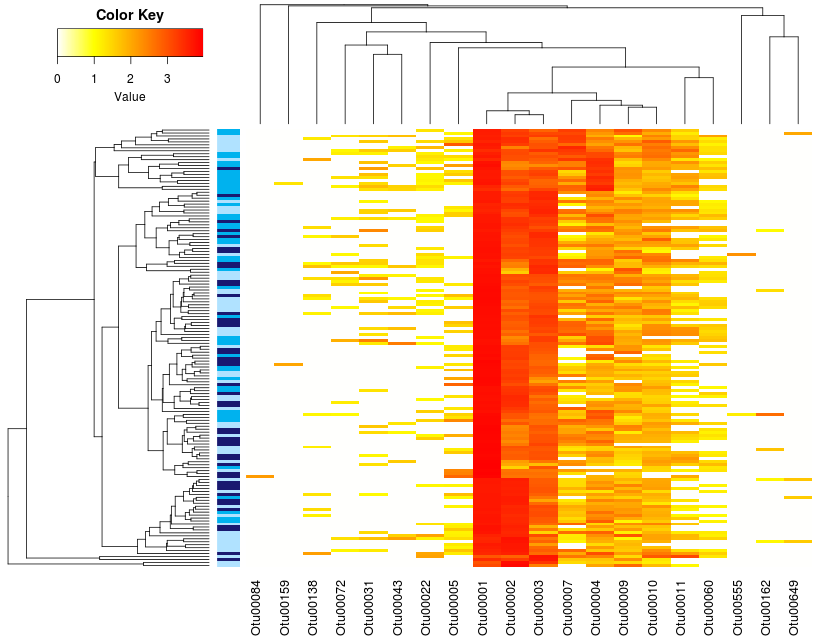
**

**Figure S3: Structure of seed-associated bacterial assemblages according to 16S rRNA gene sequences**

Hierarchical clustering of seed samples (*y* axis) is based on Bray-Curtis dissimilarity measure. The type of samples is represented by gradual color changes: light blue for controls, medium blue for seeds contaminated with *Xcc* and dark blue for seeds contaminated with *Ab*. Only abundant OTUs (threshold of 1% in relative abundance) are represented in the heatmap. These aOTUs are clustered by their co-occurrence patterns (*x* axis). According to analysis of similarity, a significant clustering of *Ab* seed samples was observed (*p* < 0.001).

**
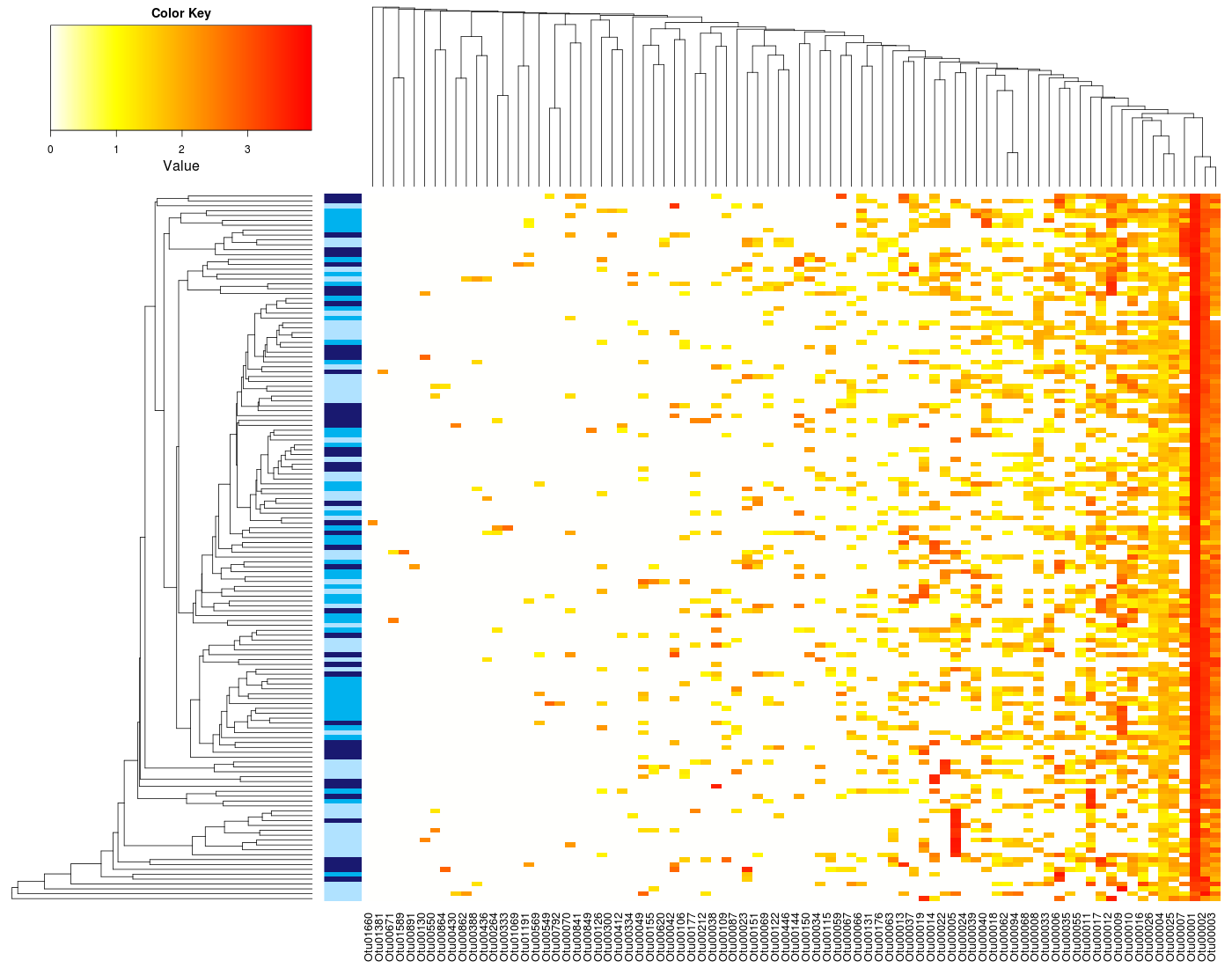
**

**Figure S4: Structure of seed-associated bacterial assemblages according to *gyrB* sequences**

Hierarchical clustering of seed samples (*y* axis) is based on Bray-Curtis dissimilarity measure. The type of samples is represented by gradual color changes: light blue for controls, medium blue for seeds contaminated with *Xcc* and dark blue for seeds contaminated with *Ab*. Only abundant OTUs (threshold of 1% in relative abundance) are represented in the heatmap. These aOTUs are clustered by their co-occurrence patterns (*x* axis). According to analysis of similarity, a significant clustering of Ab seed samples was observed (*p* < 0.001).

**
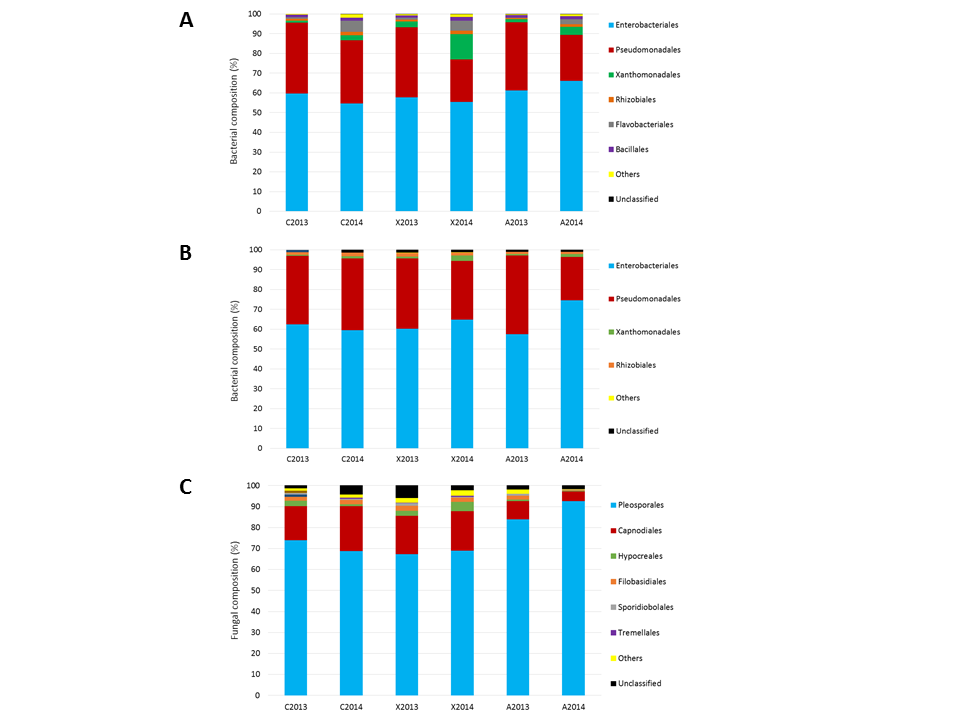
**

**Figure S5: Changes in relative abundance of microbial taxa**

Relative abundance of bacterial and fungal orders according to 16S rRNA gene (A), *gyrB* (B) and ITS1 (C). Taxonomic affiliation of bacterial aOTUs was performed with the RDP database for 16S rRNA gene, with an in-house *gyrB* database (Barret et al 2015) and with the UNITE database for ITS1.

**
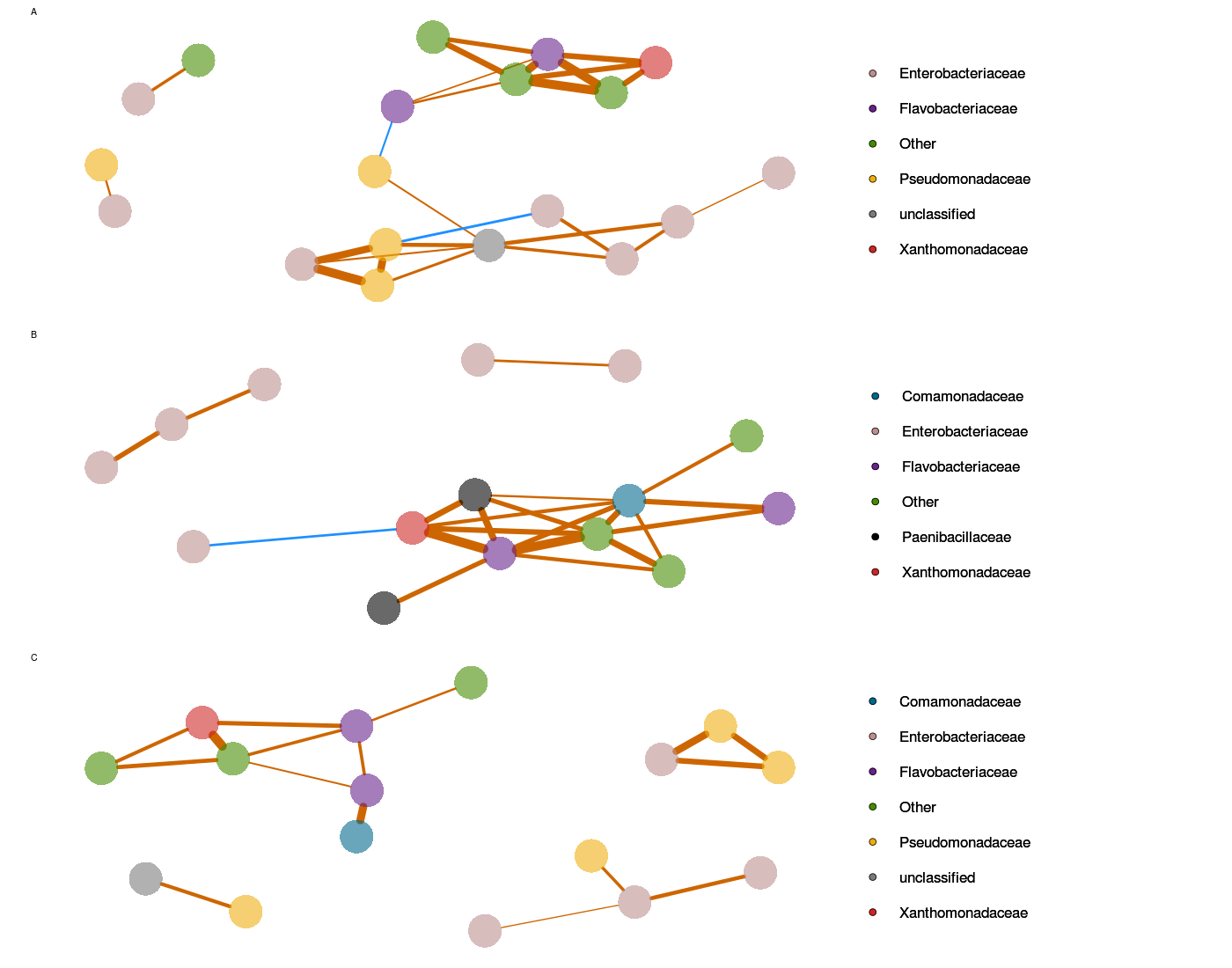
**

**Figure S6: Correlations networks between bacterial aOTUs**

Correlation networks between bacterial taxa are based on 16S rRNA gene sequences obtained in uncontaminated seeds (A), seeds contaminated with *Xcc* (B), and contaminated with *Ab* (C). Correlations between aOTUs were calculated with the Sparse Correlations for Compositional data algorithm. Each node represents an aOTUs, which is colored according to its taxonomic affiliation (family-level). Edges represent correlations between the nodes they connect with blue and orange colors indicating negative and positive inferred correlation, respectively. Only correlations with pseudo *p-*value ≤ 0.001 were represented in the network using the R package qgraph.
